# To Check or Not to Check? A Qualitative Study on How the Public Decides on Health Checks for Cardiovascular Disease Prevention

**DOI:** 10.1371/journal.pone.0159438

**Published:** 2016-07-14

**Authors:** Ai Theng Cheong, Ee Ming Khoo, Seng Fah Tong, Su May Liew

**Affiliations:** 1 Department of Primary Care Medicine, University of Malaya Primary Care Research Group, Faculty of Medicine, University of Malaya, Kuala Lumpur, Malaysia; 2 Department of Family Medicine, Faculty of Medicine and Health Sciences, Universiti Putra Malaysia, Serdang, Malaysia; 3 Department of Family Medicine, Faculty of Medicine, Universiti Kebangsaan Malaysia, Kuala Lumpur, Malaysia; Brown University, UNITED STATES

## Abstract

**Background:**

More than half of the general population does not attend screening for cardiovascular diseases (CVD) hence they are unaware of their risks. The objective of this study was to explore the views and experiences of the public in deciding to undergo health checks for CVD prevention.

**Methods:**

This was a qualitative study utilising the constructivist grounded theory approach. A total of 31 individuals aged 30 years and above from the community were sampled purposively. Eight interviews and six focus groups were involved, using a semi-structured topic guide.

**Results:**

A conceptual framework was developed to explain the public’s decision-making process on health check participation for CVD prevention. The intention to participate in health checks was influenced by the interplay between perceived relevance and the individual’s readiness to face the outcome of health checks. Health checks were deemed relevant if people perceived themselves to be at risk of CVD and there was an advantage in knowing their cardiovascular status. People were ready to face the outcome of health checks if they wanted to know the results and were prepared to deal with the subsequent management. The decision to participate in health checks was also influenced by external factors such as the views of significant others, and the accessibility and availability of resources including time and finances.

**Conclusions:**

The intention to screen for CVD is motivated by two internal factors: the perceived relevance of the disease and readiness to face screening outcomes. Strategies targeting the internal decision-making process may prove to be key in improving the uptake of screening.

## Introduction

Cardiovascular disease (CVD) remains the leading cause of death globally [1]. The number of deaths from CVD in 2015 was estimated to be 17.8 million; this represents one-third of all deaths [2]. It is the main cause of death in the middle- and high-income countries [1, 2]. Furthermore, it is predicted to be the major cause of morbidity and mortality in most of the developing countries by 2020 due to the increasing prevalence of CVD risk factors and the effects of urbanisation and lifestyle changes in these countries [3–6].

The majority of CVD can be prevented. Modelling analyses has shown that the decline in mortality due to coronary heart disease (CHD), a common manifestation of CVD, in the developed countries was largely due to a decline in cardiovascular risk factors and success in CHD treatments [7–11]. However, this decline is not seen in the developing world where the prevalence of CVD risk factors such as hypertension, diabetes and obesity is increasing at an alarming rate, and there is a lack of satisfactory control of these risk factors [12–14]. An example is Malaysia, where one-third of the adult population has hypertension and hypercholesterolaemia and one in seven has diabetes [15]. Yet, half of those with existing risk factors are unaware of their increased risk for CVD [15].

Health checks are useful for early identification of individuals at high risk of CVD [16–19]. This has prompted the implementation of national screening programmes such as the NHS Health Check in England [20] and the 45 year old health check in Australia [21]. However, the success of a prevention programme depends on the participation of the targeted groups. In Malaysia, the uptake of health checks remains low. The Malaysian National Health Morbidity Survey 2011 reported that only 37.8% of those aged 18 years and above had undergone health checks in the past year [15].

The Malaysian health care system is a dual-care system with public and private sectors. Public clinics are heavily subsidised by the Ministry of Health while private clinics charge a fee for service. In Malaysia, health checks for CVD prevention are mainly performed opportunistically by health care providers or through individual initiation. In 2013, the Malaysian Ministry of Health collaborated with the Department of Community Development and the Community Watch to implement a community-based programme to encourage the public to adopt a healthy lifestyle and undergo screening for non-communicable disease risk factors [22]. This programme is run by trained volunteers and targeted the participation of 10,000 localities, with 50,000 volunteers trained and 1.5 million adults screened for non-communicable disease risk factors. Until September 2015, 18,473 volunteers were trained in 3506 localities, and 101,875 people aged 18 years and above (6% of the targeted population) were screened. Of those screened, 12365 were referred to the nearest clinic. In addition, the Social Security Organisation (SOCSO) under the Malaysian Ministry of Human Resources provided a free one-time voucher for CVD risk assessment to all members aged 40 years and above in 2013. Despite the incentives, the uptake of this programme was only 16.2% (308309/1.9 million workers) [23].

It is therefore important to understand the public’s decision-making process to undergo health checks for CVD prevention. This study thus aimed to explore how the public decides to undergo health checks for CVD prevention and to develop an explanatory model for the decision-making process.

## Methods

### Study design

This was a qualitative study using constructivist grounded theory methodology [24]. This is a systematic yet flexible method for analysing qualitative data to construct a substantive theory to explain the phenomenon of interest [24]. The constructivist approach acknowledges the inherent subjectivity and researcher’s involvement in the interpretation of the data [24]. The phenomenon of interest in our study was the public’s decision to undergo health checks for CVD prevention. We believe that the decision is made through a cognitive process with consideration of a few factors. These factors are interrelated and their relationship is best portrayed with an explanatory model or conceptual framework, which is the expected outcome of grounded theory methodology.

### Participants

We recruited adults aged 30 years and above who lived in the Klang district and did not have a history of CVD or other serious illnesses such as cancer or psychosis. Klang is one of nine districts in the state of Selangor, Malaysia. Public and private health check facilities are widely available in this district. We recruited the above age group as this is the age group for which CVD risk screening is recommended [15]. The first three interviews and the first focus group were conducted with participants recruited through purposive sampling to include the three main ethnic groups in the country: Malay, Chinese and Indian. Recruitment was done through social network contacts. The invitation to participate was spread by word of mouth through colleagues and friends from the three ethnic groups. The inclusion criteria were explained and a patient information sheet was provided. Potential participants were then contacted and invited to participate in the study. Participants from the first four sessions (three interviews and one focus group) had undergone health checks. From the preliminary analysis and findings of these transcripts, we learnt that socio-demographic background had a great impact on health check decision-making. We then recruited, through similar methods, members of the public from different socio-economic backgrounds and those who had not undergone health checks. Participants with different characteristics were deliberately selected to challenge, refine, elaborate and exhaust the conceptual categories constructed from the initial analysis.

### Data collection

Data were collected through focus groups and interviews. Each focus group had two to seven participants who shared similar socio-demographic backgrounds and languages. A focus group provides an opportunity for participants to interact and exchange ideas to further stimulate thoughts from the interactions [25]. An interview allows participants to express their experiences in detail and voice views that they may otherwise not reveal in the presence of others [26]. All interviews and focus groups were conducted and facilitated by ATC, who is fluent in the three languages used in the community (English, Malay or Mandarin). Each interview or focus group was conducted in one of these languages as preferred by the participants. An assistant took notes during the focus groups, which were used to verify subsequent transcribing. The interviews and focus groups were carried out in participants’ homes or workplaces, whichever was convenient for them.

The participants were provided explanations about the study and written consent was obtained prior to the conducting of interviews or focus groups. A single-page data sheet was used to collect the socio-demographic data of the participants. The interviews were conducted using a semi-structured interview guide with open-ended questions (S1 Appendix). This interview guide was constructed based on a literature review incorporating the elements of the health belief model and the theory of planned behaviour model [27,28]. The questions were initially designed to explore some of the concepts of these models such as attitude and perception of susceptibility towards CVD. The use of these theories assisted in achieving interview and focus group depth. However, constant reflections were carried out to ensure openness of the interviews and focus groups. The guide was also used to assist and facilitate discussions on understanding of CVD and its risk factors, participants’ experiences and decision on health checks, and barriers and motivators for their participation in health checks. Nevertheless, participants’ opinions and experiences with cardiovascular health checks were explored without restriction from the guide. It was also not used as a framework for analysis. While the analysis and data collection were in progress, the questions in the guide were modified and new questions were generated based on findings from the earlier analysis. This allowed the researcher to examine and explore the relevance of ideas and concepts and the relationships between the concepts in subsequent interviews. The average time taken for a focus group and an interview was 74 minutes (range 60–115 minutes) and 66 minutes (range 20–81 minutes), respectively.

Data collection was continued until subsequent information did not contribute substantially to the understanding of the decision-making process.

### Analysis

All interviews and focus groups were transcribed verbatim in the original languages used. ATC reviewed and checked each transcript and referred to the field notes during the transcription process. Non-verbal cues observed during the interviews and field notes were recorded in the transcripts.

Data were organised using the qualitative data management software QSR NVivo 10 to facilitate analysis. Two researchers (ATC and SFT) read through the first three transcripts several times for data familiarisation. Next, data were analysed independently using line-by-line open coding. Subsequently, the findings were discussed and differences were debated. In the event of unresolved differences, the original transcript was revisited, discussed and new concepts were reassigned. Concepts from the three transcripts were then merged. The first three transcripts generated 733 concepts which were consolidated into categories and sub-categories. The properties of each category were defined. The transcripts and summary of the concepts were then read by EMK to examine for fitness of the concepts generated. Again, where the fitness of a concept was lacking, discussion ensued and amendments were made. ATC, SFT and EMK are all trilingual (English, Malay and Mandarin). The transcripts were transcribed in the languages used in the interviews to preserve semantics as much as possible. Coding and analysis were done in English. In the Results section, the quotes are translated into English if the language used in the transcripts was different (Refer to S2 Appendix for the original and translated quotes * presented in the Results section). Following the first three transcripts, all 11 transcripts were coded by ATC. We did not consider whether the findings were from the majority or minority of participants as we were inclusive in gathering all new findings to explain as many individuals’ thought processes for undergoing health checks as possible.

The relationships of the categories and sub-categories were explored through constant comparison of the data within and between interviews. The initial framework outlined was continually modified and reconstructed following analysis of subsequent transcripts. Frequent discussions were carried out among the researchers during data analysis. After analysing the ninth transcript, no new concepts emerged from the data and focus coding was then used. In contrast with line-by-line coding, which codes all segments of data, focus coding allows us to sieve and analyse large segments of data. Theoretical coding of causal effect was applied to construct an explanatory framework for decision-making in health checks for CVD prevention. The analysis was done through constant comparison, memoing and diagramming. Memos were used as reflexive notes for researchers to reflect and minimise the effect of preconceived ideas during analysis. Theoretical saturation was reached after analysing the ninth transcript, where no new properties of the core category were noted from the subsequent five transcripts. Feedback sessions for member checking were conducted with 18 participants (15 participants attended six group sessions, two attended individual sessions and one provided feedback via e-mail) where the results were presented. The participants were asked if the results correctly depicted their decision-making process.

This study had obtained ethical approval from the Medical Ethics Committee of the University of Malaya Medical Centre (20145–274).

## Results

A total of eight interviews and six focus groups were conducted involving 31 participants. The socio-demographic profiles of the participants are shown in Table 1. There were more women than men in the study. The interviews with the last two men recruited to the study did not add to further understanding of the decision-making process.

**Table 1:** Sociodemographic characteristics of the participants.

Characteristics	No. of participants (n = 31)
**Age range (years)**	31–60
**Age group (years)**	
30–39	3
40–49	12
50–60	16
**Gender**	
Male	7
Female	24
**Ethnicity**	
Malay	8
Chinese	13
Indian	10
**Education level**	
Primary	7
Secondary	13
Tertiary	11
**Employment status**	
Private	11
Government	9
Self-employed	2
Pensioner	1
Housewife	8
**Co-morbidities**#	
Yes	17
No	14
Experiences in health check participation*	
Frequent health check attender	17
Infrequent health check attender	9
Never attended health check	5

^#^One or more of these conditions was present: hypertension, hypercholesterolaemia, diabetes, obesity, thyroid disorders, bronchial asthma.

*Frequent health check attender: People who underwent health checks almost annually or more often than annually. Infrequent health check attender: People who underwent infrequent health checks previously and had no plans to go for a further health check.

**Fig 1:**
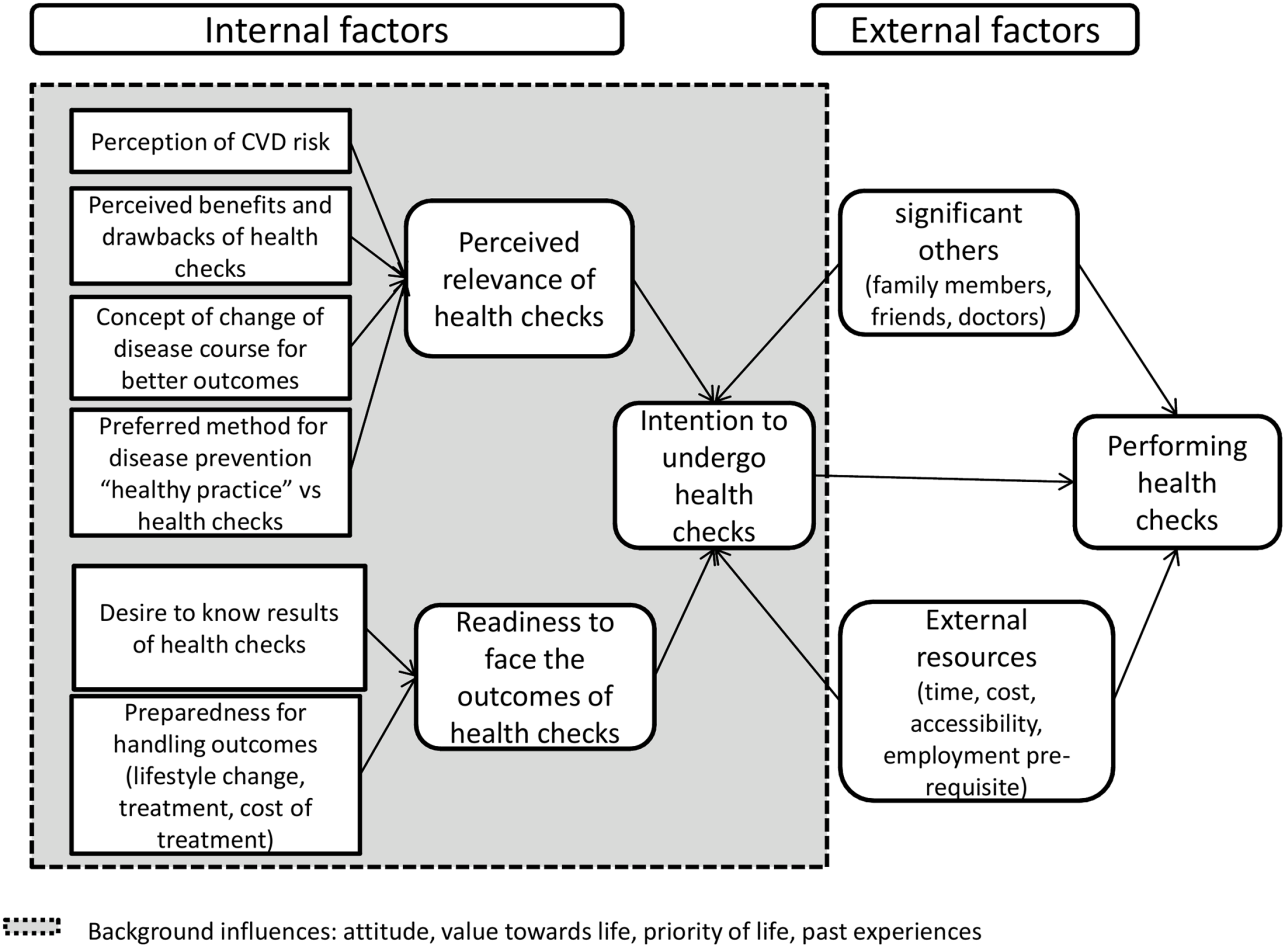
Public’s decision making process to undergo health checks for cardiovascular disease prevention.

The main factor in people deciding to undergo CVD prevention health checks was the degree of intention for participation, which resulted from the interplay between perceived relevance of health checks and readiness to face the outcomes of health checks. In addition, external factors, such as the influence of significant others, and resources, such as accessibility to health care facilities, time, venue of the health checks, and cost of screening tests could modify the intention and realisation of undergoing health checks. Fig 1 illustrates the conceptual framework of the decision-making process in health checks participation.

### Perceived relevance of health checks

The public’s perception of the relevance of health checks was a net gain in health benefits from undergoing health checks. For health checks to be seen as relevant, individuals would have to perceive themselves to be at risk of CVD and have the intention of improving their chances of a better outcome. Health checks would be perceived as a preferred strategy in disease prevention in addition to lifestyle modification or/and use of complementary medicine.

#### Perception of CVD risk

The perception of being vulnerable to CVD depended on an individual’s stance on CVD risk factors, such as increasing age, presence of family history, unhealthy lifestyle and presence of co-morbidities such as hypertension and hypercholesterolaemia. The use of ‘I’ in conversation denoted internalisation of the risks.

‘I personally think there is a need for it [health checks], especially my age is older, 50-odd years old, so I felt I should have, earlier check…’* 56-year-old Chinese woman, teacher, frequent health check attender, focus group

The perceived risks of CVD differed between age groups. Older people viewed age as a CVD risk factor, but younger people viewed a stressful lifestyle as a risk.

‘If for me *lah*, it [the risk] is more on stress. When stress, you can easily get hypertension. If you have hypertension, of course it is easy for you to have heart problem.’* 33-year-old Malay woman, receptionist, infrequent health check attender, interview

On the other hand, aging may not be viewed as a CVD risk but rather as a natural process of life.

‘No, I think this [father’s history of heart attack] is nothing to do with me, because they are old, they would have it [heart attack]. Like my mum, she has… high blood pressure, diabetes, she has everything, everything.’* 54-year-old Chinese man, contractor, never attended health checks, interview

People also felt the need for CVD checks if they had symptoms or had chronic diseases. Otherwise, they did not feel the necessity for health checks.

‘Why don’t I want to go [health checks] *ah*? I have nothing wrong [no symptoms], why do I need to go? I think I have nothing wrong, why bother going?’* 49-year-old Chinese woman, school bus driver, infrequent health check attender, focus group

‘That is, that is because of my neck swelling [thyroid]… at that time I went to see the doctor, since then I began to go for checks every year… So, it’s because of my neck swelling, it’s like this, I will continue like this to go for body check.’* 42-year-old Chinese man, supervisor, frequent health check attender, interview

The relationship between perceived health checks relevance and perceived risk of CVD was not straightforward. The relevance of health checks depended on the perceived risk for CVD and the weight assigned to the risks. For example, in one focus group, a participant felt that she was at risk for CVD due to her lifestyle, but did not feel the urge to undergo health checks as she did not experience any discomfort. Thus, having symptoms was perceived as more relevant for health checks than having a CVD-risky lifestyle.

‘I think yes [have the risk of getting CVD], because our eating habits, our environment is not so healthy, so I think there is [risk]… The main problem is, the main problem is [to go for health checks], I should say, it’s because there is no serious problem encountered yet.’* 48-year-old Chinese woman, teacher, infrequent health check attender, focus group

The degree of relevance varied with circumstances. The perception of risk could be enhanced by the occurrence of a significant event, such as death among friends, which served as an impetus for the relevance of health checks and participation therein.

‘We both talk about it and… most of our friends are like 40, 42, and 45. Sometimes they passed away you know. Sometimes we are so shock, heart attack, so *ah* we must, *errr*, better do. So my husband said, wow we better do it [health check].’ 50-year-old Malay woman, housewife, never attended health checks but plans to attend, interview

#### Perceived benefits and drawbacks of health checks and possibility of a change in the course of CVD outcome

The perceived relevance of health checks depended on how individuals weighed its benefits and drawbacks. The benefits considered included personal awareness of health status, sense of security in terms of health, early detection of risk factors and diseases and the opportunity for disease prevention and treatment. These benefits are linked closely with the idea of diseases and a possibility of changing the CVD course and outcomes if changes such as lifestyle modification to the identified risk factors were made.

‘…my rationale for blood test, I think… if the disease has happened to me, actually I can’t do anything… So, it is better to know earlier than later. If I know early I can decide what to do, I can still do something about it, isn’t it? If know it later, then it’ll all be left to fate…’* 47-year-old Chinese woman, teacher, frequent health check attender, focus group

On the contrary, if an individual believed that life was predestined and there would be no possibility of changing the course of CVD outcome, and they would not undergo health checks. In addition, knowing the limitation of testing, where a normal blood test does not rule out the presence of disease, and the inconvenience of blood-drawing and the process of having to fast for blood-drawing were barriers to undergoing health checks.

‘…you go to see specialist *lah*. I heard from my friend, nearly a hundred plus ringgits *lah*, medical checkup *lah*, everything *lah*, give you to know, answer everything (you) got, but what for, if you know, how? …Don’t waste your time, you can eat how much, drink how much, it’s given by God. It’s true. I don’t feel shameful to say this. Seriously I tell you, it’s better not to think too far ahead *lah*. Let’s live day by day. If you can’t live beyond the day, that’s decided by the god, not you …He [the god] wants you to die; you can’t avoid death, isn’t it?’* 54-year-old Chinese, contractor, never attended health checks, interview

#### Preferred method for disease prevention: ‘healthy practice’ vs. health checks

Some participants preferred adopting healthy lifestyle practices such as sleeping well, exercising, following a healthy diet, reducing stress, or using alternative medicine such as qi gong and tai chi over health checks for diseases prevention.

‘For me, I am very reluctant to do health checks. Because I know this, our body has the ability to heal on its own, and this is my preferred way. Not going for health checks… we have learned what he said about “longevity studies”. We believed this will slow down the degenerative process, then it made the body function rejuvenates… this is what I liked the most, so I’ll learn this.’* 48-year-old Chinese woman, teacher, infrequent health checks attender, focus group

Others believed that undergoing regular health checks would inform personal health status and prevent disease.

‘Annual is just blood pressure, no I mean blood test. Then for me to wait for the next year will be too late. Better, I mean, I have a frequent check. Anything I can prevention earlier.’ 50-year-old Malay woman, senior hospitality manager, frequent health check attender, interview

### Readiness in facing health checks outcomes

Readiness to face the outcomes of health checks refers to a person’s mental preparedness to deal with health checks outcomes such as the results, diagnosis, the need for medication or lifestyle modification and the cost incurred from management following health checks. The stage of readiness to face health checks outcomes influenced a person’s intention to participate in health checks.

‘…you put it [abnormal results] under (the) table, it is better not to let me know. If it is a little [abnormal]… ah, that is ok. I think it is better not to let me know, because we will not feel the pressure then, need to take medicine, headache [stressful], we have work to do. Oh. Then, we have to do this and that [have to follow the advice in disease management]. Some medicines cause sleepiness, right? That’s why, we have to work, we cannot [afford to fall asleep] … let me tell you, you have to standby [be prepared], if the doctor tells you, what your problem is, you must accept, that’s all. Go for health checks, you must accept [the outcomes], believe. If you do not believe, don’t go.’* 54-year-old Chinese man, contractor, never attended health checks, interview

An infrequent attender of health checks was not ready for treatment because he did not believe he had the disease and did not want to be treated.

‘After seeing the doctor, gave me medicine. I follow *lah*. It should be every day. But sometimes I take it today, tomorrow I don’t. I am lazy to think about this, I think I don’t have disease.’* 52-year-old Malay man, administrative assistance, infrequent health check attender, interview

### Background influences

Background influences are other factors that have indirect roles that influence the intention to undergo health checks (Fig 1). These included attitude, personal values towards health, life priority, life goals and life experiences. These factors could affect a person’s perception of the net benefit of health checks (perception of the relevance of health checks) and readiness to face the outcomes of health checks. For example, a middle-aged woman who had a life goal of caring for and raising her young children valued the importance of health for her to carry out this responsibility. This had encouraged her to take care of her own health and thus she felt that health checks are beneficial and was ready to accept subsequent management following health checks to maintain her health.

‘…because our children were still young. We thought, *aiyo*, how are we going to look after them, we have to take care of ourselves first, then we can see them growing up. If the two of us are not around [die], like what you said, getting stroke, or getting whatever, who is going to look after them?’* 54-year-old Chinese woman, housewife, frequent health check attender, focus group

### External factors

The intention and decision to undergo health checks could be influenced by external factors such as the views of significant others, accessibility of health checks, convenience of time and venue for health checks, and cost. Some people had the strong intention of undergoing health checks, and they would do so regardless of external factors.

‘No [appointment], about a year I will go for checks. I also keep the report *lah*. But the doctor says do not to waste money. He said you come around one and a half years; I said never mind, never mind, I will go for checks.’* 34-year-old Chinese man, labourer, frequent health check attender, focus group

Some participants who had weaker intention for undergoing health checks would participate if the health checks were readily available, accessible, convenient and if they had peer accompaniment.

‘Actually, I was afraid to face these sorts of things, blood taking, the clinic and the smell, I am actually very, very frighten of these. So when he [health checks team] came here, I asked my colleagues whether they want to go together for checks. Because for me, I don’t go for yearly check, although I know it is best to do it once a year, but I thought I won’t bother to go. Now that he is here, and there are colleagues here, must go together [with colleagues], to take blood, ha ha, then I will go just this.’* 48-year-old Chinese woman, teacher, infrequent health check attender, focus group

‘If there is a promotion, there is a package [promotion] I will go for checks directly.’* 46-year-old Chinese, teacher, infrequent health check attender, focus group

Nevertheless, for some participants, accessible and free health checks would not persuade them to attend the checks. A participant who had been given a free voucher did not utilise this privilege as she believed that her current health was unproblematic.

‘No [not going for health checks]. I do not feel anything [symptoms], I feel there is no problem. I did not go [for health checks].’* 43-year-old Malay woman, kindergarten headmistress, never attended health checks, focus group

### Validation of results

The concepts generated from the interviews were fed back to the participants. All participants agreed with the concepts presented in the feedback sessions.

## Discussion

### Summary of findings

We had developed a conceptual framework to provide an overview of the decision-making process in the participation of CVD prevention. The decision to undergo CVD health checks was multi-factorial. The main factor was an individual’s intention to undergo health checks, which was a result of the perception of relevance and the state of readiness to act on or cope with the findings of the health checks. Influences from significant others, as well as time, cost, accessibility and health care facilities also contributed to the decision to undergo health checks. Each decision made to undergo a health check also depended on the weightage placed on the various components of the framework. For example, previous health checks experiences influenced the weightage placed on subsequent decisions to undergo health checks.

### Comparison with other studies

While other studies have focused on exploring patients’ influences, barriers and motivators to participate in health checks [29–33], we developed a framework to explain how the decision-making process was formed for health checks. The framework illustrates a dynamic process of decision-making for health check participation. It also illustrates the complex interplay within and between each concept. For example, the external factors for decision-making on CVD prevention health checks such as time, cost and accessibility were barriers for health checks participation in the literature [29,31,34]; however, further exploration of the relationship between these factors has shown that the extent of the barrier is relative to the intention to undergo health checks.

Individual who perceive health checks as relevant and are ready to handle the outcomes of health checks would have a greater intention to undergo health checks and be less influenced by external factors. On the other hand, individuals with low intention for health checks might not attend despite having favourable resources. This was illustrated by the low uptake rate of the SOCSO health screening programme in Malaysia (16%) [23] despite members being provided a free voucher and convenient access to health checks at their chosen clinics. Similar results have also been reported elsewhere, where the response rate to free health checks conducted within working hours near the workplace for low-paid government employees in England was only 20% [35]. Thus, the intention to undergo health checks is apparently determined prior to be being influenced by external factors.

The perception of CVD risk, the possibility of a change in CVD outcomes and the perceived benefit of health checks, which contribute to the perception of the relevance of health checks, are also addressed by other health behaviour theories, such as the health belief model and the theory of planned behaviour model [27,28,36]. The health belief model predicts that individuals are more likely to take action if they perceive themselves to be susceptible and believe their actions will reduce susceptibility and minimise the consequences of the problem [27]. Our findings concur with this, where individuals who underwent health checks were those who perceived themselves to be at risk of CVD, perceived health checks as beneficial for early detection and treatment and believed in the possibility of CVD prevention and treatment. In the planned behaviour theory, individual behaviour is influenced by attitude. For example, a positive belief and attitude toward health checks such as the perceived benefits of health checks and early treatment would promote the intention to undergo health checks [28]. We also found that significant others such as doctors, friends, spouse or relatives could influence intention for health check participation and these social influences resemble the subjective norm in the planned behaviour theory, which could motivate or demotivate an individual to undergo health check [28].

Another important concept is the readiness to face the outcomes of health checks. People avoided knowing their CVD risk due to the fear of identifying health problems and the consequences of health checks [29,31,32]. These fears are indicative of the lack of readiness to face the outcomes of health checks and hence weakens the intention to undergo health checks. The readiness to face the outcomes of CVD health checks is similar to the readiness for change in many health interventions, such as quitting smoking and treating obesity, where the 5As (ask, assess, advise, agree and assist) approach [37,38], and Prochaska’s trans-theoretical model [39] are used. A similar model could perhaps be used in the future to assess readiness for health check participation.

Another reason to undergo health checks is when an individual is symptomatic and perceives that they are at risk of CVD, portraying a reactive, help-seeking behaviour [29,31,33]. This also suggests a lack of understanding of screening and disease prevention, a deterrent in CVD health checks participation. We also found that some individuals opted for healthy practices such as getting adequate sleep, exercise, and avoiding stress over health checks for disease prevention and health maintenance; which is consistent with other literature [33].

### Recommendations/implications

In order to improve the uptake rate of CVD prevention, a multi-faceted approach addressing intention to participate, perception of relevance, and state of readiness to act on or cope with health checks findings, as well as having favourable external factors (flexible appointment times, cost, and accessibility) is needed.

Several measures could be employed to improve public perception of health checks relevance. One measure is to improve the understanding of CVD prevention and the perceived CVD risks. This can be done by incorporating personalised risk communication in health checks invitations [29, 31]. For example, stratification of CVD risk according to age, ethnicity and presence or absence of CVD risk factors can be made known to the public. This will allow members of the public to understand their own risks for CVD. During clinical consultation, health care providers could also discuss personalised CVD risk scores with patients. In addition, any intervention to promote CVD risk prevention needs to take into consideration life goals, life priorities, family and work commitments, which could play a part in decision-making.

The autonomy of individuals who opt for healthy practices such as healthy diet and physical activity over health checks for disease prevention and health maintenance should be respected [33]. Health care providers could facilitate informed decision-making by the public through education and dissemination of the evidence and effectiveness of such practices.

The lack of readiness to face health checks outcomes could result in non-participation in health checks and failure in subsequent management. As a health check is the first step in a health intervention, intervening at this point might benefit subsequent management and optimise the benefits of health checks. A previous local study reported the decision of physicians to engage patients in health checks based on the perceived balance between patient receptivity and the relevance of the issues to the patient [40]. However, patients might not be receptive if they are unprepared to face health check outcomes. Therefore, to encourage engagement in health checks, health care providers need to assess and address public readiness for health checks objectively and provide support. For example, for opportunistic health check invitations, a prompt sheet could be provided to potential participants to determine whether they have any concerns about the health check which can be addressed during consultation. When disseminating information to the general public, common issues such as the side effects of medication, health check procedures or lifestyle management could be included to reduce misconceptions and enhance readiness to face the outcomes of the health checks.

### Strength and limitations

This study recruited members of the public with a wide spectrum of health checks experiences (those who were committed to regular health checks, those who had attempted but did not sustain health check activities and those who had never gone for health checks). The framework was grounded in the data and was relevant and fitted the explanations of the participants’ decision-making processes. Besides validating barriers and motivators noted in many studies concerning health checks and help-seeking behaviour [29,31–34], the framework explained the relationships between the concepts derived from the empirical data on the topic of understanding of CVD and its risks and health check practices on CVD prevention. Its transferability to another setting will require further testing. Our participants consisted of three major ethnic groups and were from a district where health care facilities are widely available. The views from other minority ethnic groups and those from deprived areas, such as the indigenous groups, were not covered. Thus, the framework might not be able to explain the decision-making process of these populations.

## Conclusion

The decision to undergo health checks for CVD prevention depends on an individual’s intention to participate, perception of relevance and readiness to accept or cope with health checks findings. Interventions to encourage participation in health checks need to address these issues.

## Supporting Information

S1 Appendix Interview Topic Guide. (DOCX)

S2 Appendix Original quotes presented in the result section. (DOCX)
