# Correction: To Check or Not to Check? A Qualitative Study on How the Public Decides on Health Checks for Cardiovascular Disease Prevention

**DOI:** 10.1371/journal.pone.0162152

**Published:** 2016-08-25

**Authors:** Ai Theng Cheong, Ee Ming Khoo, Seng Fah Tong, Su May Liew

The affiliation for the fourth author is incorrect. Su May Liew is not affiliated with #3 but with #1 Department of Primary Care Medicine, University of Malaya Primary Care Research Group, Faculty of Medicine, University of Malaya, Kuala Lumpur, Malaysia.
