# Supplementary material for: To Check or Not to Check? A Qualitative Study on How the Public Decides on Health Checks for Cardiovascular Disease Prevention
Source: PLoS One. 2016 Jul 14;11(7):e0159438. doi: 10.1371/journal.pone.0159438 (PMC4945067; doi:10.1371/journal.pone.0159438)
Supplement: S1 Appendix — (DOCX) [file pone.0159438.s001.docx]

**S1 Appendix. Interview Topic Guide**

**Introduction**:

- Ice-breaking
- Explain the objectives of this study and no right or wrong answer
- Do not have to answer if the participant doesn’t wish to do so
- Reinforce on confidentiality
- Get consent for the interview and audio-recording
- Participants to fill up the participant’s background questionnaire before interview

**Triggers for discussion:**

1. Have you heard about cardiovascular diseases such as stroke and heart attack? What do you think about these diseases? What’s the impact? Any experiences (own relatives or others)? (if yes, tell me more about the experiences).
2. What lead to stroke? What lead to heart attack? How much you think this risk factor (the factor mention by participants eg hypertension) lead to heart attack or stroke?

1. Who are the people you think will get heart attack/stroke? Why? Do you think you are at risk? Why?
2. Doctor sometimes tells the patient about their risk to develop heart/stroke in 10 years.

What do you understand by having a heart attack/stroke risks in 10 years time?

Doctor usually quantify the risk in 100 people in 10 years and they will put it as low, moderate or high. What do you consider to be high risk?

(probe: Some say 10, some say 50,70% , how about you?)

(High risk 20 in 100 people (20%) will get the heart attack in 10 years.)

1. Have you heard about health check-up/medical check-up? What do you understand by the term health check up/medical check-up?
2. Have you gone for the check up? Tell me your experience

(probe: When is the last health check-up? Where did you have it done? What is it for (cardiovascular diseases/ cancer prevention/job requirement etc? What were the things/tests you have undergone? What were the results?)

1. How this came to your mind? What made you to/not to take up the check-up at that time? Why (reasons for going and not going)?
2. Do you know someone who do not go/go (with opposite behavior) and why they do so? Are these (reasons given) applied to you?
3. Do you think it is useful? Why?
4. Have you heard about health check-up to prevent heart attack/stroke? What do you understand about this check-up? Have you gone for it? Tell me your experience (if no.6 not cover this).
5. Any problems you are worry about for doing health check-up?
6. Has anyone/any doctor/nurses ever tell you about your risk in getting heart attack? What did they say (informed by using risk score)? What do you think of that?

13. Have you ever been invited for health check-up for prevention of stroke/heart attack? Where was the invitation from? How do you response?

1. Do you think the doctor should encourage people to come for health check? If the doctor /health care professional invite you for health check-up for prevention of stroke/heart attack, what will make you acceptable? (Would you and why?)
2. How do you think the clinic or doctors can do to encourage people to come for health check up?
3. Anything else you would like to tell me?
