# Supplementary material for: To Check or Not to Check? A Qualitative Study on How the Public Decides on Health Checks for Cardiovascular Disease Prevention
Source: PLoS One. 2016 Jul 14;11(7):e0159438. doi: 10.1371/journal.pone.0159438 (PMC4945067; doi:10.1371/journal.pone.0159438)
Supplement: S2 Appendix — (DOCX) [file pone.0159438.s002.docx]

S2 Appendix. Original quotes presented in the result section

| Original quotes | Translated quotes |
| --- | --- |
| *“我个人是觉得是一种需要啦，特别是我的年龄比较大吗，五十多岁了，所以觉得应该要有，早一点check，…………..。另一方面因为我妈妈本身有高血压的，then我的哥哥他们也是有高血压，我阿姨他们都有，阿姨舅舅. So far是还好啦，我因为我的血压是偏低的，每次check我的血压都是偏低的，我不懂会不会偏低的会跑到高血压去我就不知道啦，但是就是on safer side我都是会check啦…………………….”* | *“I personally think there is a need for it [health checks], especially my age is older, fifty odd years old, so I felt I should have, earlier check, …………. On the other hand my mother has high blood pressure, then my brothers also have high blood pressure, my aunts also have, aunts and uncles. So far is okay, I, because my blood pressure tended to be low. Every time when my blood pressure is checked it is low. I do not know low blood pressure will turn into high blood pressure, I do not know. But on safer side I will always check lah………….”* |
| *“Kalau for me lah, memang more on stress. Bila stress, you akan mudah untuk kena hypertension. Bila hypertension, memang mudah untuk you punya jantung ada masalah. For me lah untuk I punya lifestyle untuk I punya kehidupan macam ni lah ……..…………… Tapi saya tahu, kalau saya punya masakan, meal, drinks semua less sugar, less salt, saya tak rasa itu akan sumbangkan benda tu dekat saya. Mungkin, kalau I punya sendiri, mungkin I perlu control, I punya stress only, because I sometimes kadang-kadang tak boleh tahan dengan sudah tired kerja, lagi nak anak, lagi nak teach dia, and then nak kena handle dia semua, itu yang kadang-kadang stress sangat lah.”* | *“If for me lah, it [the risk] is more on stress. When stress, you can easily get hypertension. If you have hypertension, of course it is easy for you to have heart problem. For me it's the lifestyle, it’s my life ............... But I know, if my cooking, meal, drinks , all have less sugar, less salt, I do not think it bringing on the risk to me. Maybe for me , maybe I need to control, my stress only, because I sometimes cannot cope with work, tired from work, and children, and need to teach him, and have to handle him and everything, these are sometimes very stressful .”* |
| *“没有，我认为这个[father’s history of heart attack] 不管我的事，因为他们老了应该有的。好像我妈咪，她有…血压高啊，糖尿啊，全部有，她全部有。”* | *“No, I think this [father's history of heart attack] is nothing to do with me, because they are old, they would have it [heart attack]. Like my mum, she has…. high blood pressure, diabetes, she has everything, everything.”* |
| *“为什么我不要去啊？都没有事情啊，去什么? 我觉得没有事情啊，去来做么?”* | *“Why don’t I want to go [health checks] ah? I have nothing wrong [no symptoms], why do I need to go? I think I have nothing wrong, why bother going?”* |
| *“就是，就是我的“大颈包”（Cantonese-thyroid）引起………..那时候去看了医生就开始每一年都去check了咯。因为我的“大颈包”是每一年都要去看它有没有继续在，在好像又大粒，变化很大啊，什么这样咯。每一年都要去看咯，就是看了就是全身检查到完。就是那个“大颈包”就是这样咯，会继续下去这样去body check咯.”* | *"That is, that is because of my neck swelling [thyroid]......... ..at that time I went to see the doctor, since then I began to go for checks every year. Because I need to have follow up on my neck swelling every year to see if it is still there, getting bigger, any changes, something like this. . Every year I need to go for follow up so I will then do check up for the whole body too. So, it’s because of my neck swelling, it’s like this, I will continue like this to go for body check.”* |
| *“我觉得是有的[有中这个心脏和中风的风险]，因为我们的饮食生活，我们的环境都不是那么健康，所以我觉得是有…….主要的难处啊，主要的难处就是说，应该是还没有遇到很严重的问题，所以就是觉得（xxx: 健康）还可以的话就是得过且过这样….”* | *“I think yes [have the risk of getting CVD], because our eating habits, our environment is not so healthy, so I think there is [risk]…….. The main problem is, the main problem is [to go for health checks], I should say, it’s because there is no serious problem encountered yet. so I just felt (xxx: healthy), if it is still fine, then let it be …”* |
| *“….但是我觉得，我验血的心态啦，我觉得啦，我验血的心态我会觉得，如果那个疾病找上我，其实我也是没有办法的，因为它在里面，我要怎样知道? 所以，这样早知道好过迟知道啦，这样早知道我还能够做主嘛，我还能够做些什么不是啊，迟知道就只好听天由命。………”* | *“... But I think, my rationale for blood test, I think, I think I go for blood test, if the disease has happened to me. Actually I can’t do anything, because it is in the body, how do I know about this? So, it is better to know earlier than later. If I know early I can decide what to do, I can still do something about it, isn’t it? If know it later, then it’ll all be left to fate. .........”* |
| *“….你去看specialist 啦，你去看啦，我听我的朋友讲，百多块啦，medical checkup啦，everything啦，give you to know，answer everything got，but what for，if you know, how？you can sleep or not? You cannot sleep, what for? Don’t waste your time, you can…eat how much,drink how much,tuhan kasi punya，betul punya，saya cakap tak ada malu punya。真的我跟你讲，做人是比较不要想那么远啦，今天能过就今天过，不能过是天给的嘛不是你要的嘛 ，……..他要你死，你不能不死的嘛对不对？对咯，…”* | *“….you go to see specialist lah, you go to see lah. I heard from my friend, nearly a hundred plus ringgits lah, medical checkup lah，everything lah，give you to know，answer everything (you) got, but what for, if you know, how? You can sleep or not? You cannot sleep, what for? Don’t waste your time, you can… eat how much, drink how much, it’s given by God. It’s true. I don’t feel shameful to say this. Seriously I tell you, it is better not to think too far ahead lah. Let’s live day by day. If you can’t live beyond the day, that’s decided by the god, not you …… He [the god] wants you to die; you can’t avoid death, isn’t it? Right…* |
| *“ 如果给我的话啦，检查对我来讲我是很抗拒的哦，如果给我的话，因为以前我就知道了，从报章上啊，或者是一些专家所讲的，那一个书啊，然后就是说我们身体本身有那个自愈的能力，然后呢，我最喜欢的就是这类型，不要去检查那一类型的，所以好像以我跟xxx啦，2013年尾我们就学到那种他说比较长生学的，我们是认为他会使到你慢一点退化，这样你身体的那些本来有的功能就让它活化起来，这一些就是我最喜欢的咯，所以我会去找这样子的来学.”* | *“For me, I am very reluctant to do health checks, if for me. Because I know this, previously, from the newspaper, or talks by some experts, books, that our body has the ability to heal on its own, and this is my preferred way. Not going for health checks. So it’s like what I mentioned to xxx, at the end of 2013, we have learned what he said about “longevity studies”. We believed this will slow down the degenerative process, then it made the body function rejuvenates ……, this is what I liked the most, so I’ll learn this.”* |
| *“……..你跟我放under table，不要给我知道比较好咯。有一点点…啊ok，好像你不要给我们知道比较好，因为我们不会去压力吖，要吃药，头痛，要做工哦。又要怎样怎样. 有的药吃了要睡觉的对不对？所以咯，我们要做工的，不能…….……我跟你讲了，要自己standby，如果医生跟你讲，你有什么问题，你要接受，一样罢了。要去看[health checks]一定要接受。Believe，你不believe叻不要去看，不要。”* | *“..... .. you put it [abnormal results] under (the) table , it is better not to let me know. If it is a little [abnormal]... ah, that is ok. I think it is better not to let me know, because we will not feel the pressure then, need to take medicine, headache [stressful], we have to work oh. Then, we have to do this and that [have to follow advice in disease management]. Some medicines cause sleepiness, right? That’s why, we have to work, we cannot [afford to fall asleep].......let me tell you, you have to standby [be prepared], if the doctor tells you, what your problem is, you must accept, that’s all. Go for health checks, you must accept [the outcomes]. Believe, if you do not believe, don’t go.”* |
| *“Bila jumpa doktor balik tu, bagi ubat yang saya, you ada ubat lagi? Ada. Dia bekalkan, saya ambil following la. It should be every day. Tapi kadang saya ambil hari ni saya, besok saya tak. Saya malas nak fikir, saya fikir diri saya tak ada penyakit.”* | *“After seeing the doctor, gave me medicine. Do you have medicine? Yes. He supplied it [medicine]. I follow lah. It should be every day. But sometimes I take it today, tomorrow I don’t. I am lazy to think about this, I think I don’t have disease.”* |
| *“Kita kena tahu juga status diri kita ni kat mana. Maksud saya blood pressure kita berapa, kita punya sugar in the blood berapa kan, kolesterol berapa. After that kalau perlu kita control. Kalau ok kita maintain. Bagi saya la. Saya punya pandangan macam tu la. Kita macam tak boleh ambil mudah, whatever nak jadi, jadi kan. Tiba-tiba collapse, cannot help already. Memang kena tahulah status diri kita…….”* | *"We need to know what the status of our health is. I mean what our blood pressure is reading, what is the sugar level in the blood, the cholesterol level. After that, if needed we will control. If it is ok, we will maintain. For me lah. My view is like that lah. We cannot take things for granted, whatever is going to happen, it will happen. Suddenly [if we] collapse, cannot help already. Indeed, we need to know our own [health] status…... "* |
| *“...四十五岁以上咯，那时我老公也是五十多岁了的，我们两个也是孩子还小，又想到，唉哟，以后要怎样子去看他们叻，我们也要照顾我们自己先嘛，才可以看他们长大嘛。如果我们两个沒有了，像你讲，等下中风啦，中什么，谁去看他们？……….”* | *"... more than forty-five years old [started to take up health checks], at that time my husband was fifty odd years old. We also, because our children were still young. We thought, aiyo, how are we going to look after them, we have to take care of ourselves first, then we can see them growing up. If the two of us are not around [die], like what you said, getting stroke, or getting whatever, who is going to look after them? .......... "* |
| *“没有[appointment]，我看大概要一年了这样，去验咯，report也是有收住啦. 但是医生讲呢，不用浪费钱啦，他讲你一年半这样才来啦，我讲不用紧不用紧，去验.”* | *“No [appointment], about a year I will go for checks. I also keep the report lah. But the doctor says do not to waste money. He said you come around one and a half years; I said never mind, never mind, I will go for checks."* |
| *“其实我是很怕面对这些的，抽血啊那些啊，去到那个药房那种药味，我其实是很怕很怕的那种，所以他来到这边，就问同事要不要，走咯，我们去检查咯，反正我，因为我，反正就不会检查的，就是说没有所谓的一年一次，虽然我知道说最好一年一次啦，可是我说，哎哟，我没有去就没有去啦，他来到这边，有同事一起，****还要一起的哦****，去给他抽血（哈哈）所以我才去咯，就是这样咯.”* | *“Actually, I was afraid to face these sorts of things, blood taking, the clinic and the smell, I am actually very, very frighten of these. So when he [health checks team] came here, I asked my colleagues whether they want to go together for checks. Because for me, I don’t go for yearly check, although I know it is best to do it once a year, but I thought I won’t bother to go. Now that he is here, and there are colleagues here,* ***must go together (with colleagues)****, to take blood (ha ha), then I will go just this”* |
| *“如果是有promotion的，有package的我就直接去验咯”* | *“If there is a promotion, there is a package [promotion] I will go for checks directly.”* |
| *“Tak ada. Saya pun tak ada rasa apa-apa, rasa macam tak ada masalah. Tak ada pergi.”* | *"No [not going for health checks]. I do not feel anything [symptoms], I feel there is no problem. I did not go [for health checks]."* |
| *“ 同意, 她讲的时候, 我就想，我是属于这一类的。.” 48 year-old-Chinese, teacher, FGD*  *“ 很完整哦.” 46 year-old-Chinese, teacher, FGD*  *“ 讲中我们。” 56 year-old-Chinese, teacher, FGD* | *“Agree [with the results presented], when she [the researcher] presented the results, I thought, I belonged to this category.” 48 year-old-Chinese, teacher, FGD*    *“Oh, it’s very complete [the results].” 46 year-old-Chinese, teacher, FGD*  *“These [results] reflect what we are.” 56 year-old-Chinese, teacher, FGD* |
